# Neurotrophic factor changes in the rat thick skin following chronic constriction injury of the sciatic nerve

**DOI:** 10.1186/1744-8069-8-1

**Published:** 2012-01-10

**Authors:** Jennifer C Peleshok, Alfredo Ribeiro-da-Silva

**Affiliations:** 1 Department of Pharmacology and Therapeutics, McGill University, Montreal, Quebec H3G 1Y6, Canada; 2 Alan Edwards Centre for Research on Pain, McGill University, Montreal, Quebec H3A 2B2, Canada; 3 Departments of Anatomy and Cell Biology, McGill University, Montreal, Quebec H3A 2B2, Canada

**Keywords:** sciatic nerve, nerve growth factor, chronic constriction injury, mast cell, peptidergic, p75, Schwann cell

## Abstract

**Background:**

Cutaneous peripheral neuropathies have been associated with changes of the sensory fiber innervation in the dermis and epidermis. These changes are mediated in part by the increase in local expression of trophic factors. Increase in target tissue nerve growth factor has been implicated in the promotion of peptidergic afferent and sympathetic efferent sprouting following nerve injury. The primary source of nerve growth factor is cells found in the target tissue, namely the skin. Recent evidence regarding the release and extracellular maturation of nerve growth factor indicate that it is produced in its precursor form and matured in the extracellular space. It is our hypothesis that the precursor form of nerve growth factor should be detectable in those cell types producing it. To date, limitations in available immunohistochemical tools have restricted efforts in obtaining an accurate distribution of nerve growth factor in the skin of naïve animals and those with neuropathic pain lesions. It is the objective of this study to delineate the distribution of the precursor form of nerve growth factor to those cell types expressing it, as well as to describe its distribution with respect to those nerve fibers responsive to it.

**Results:**

We observed a decrease in peptidergic fiber innervation at 1 week after the application of a chronic constriction injury (CCI) to the sciatic nerve, followed by a recovery, correlating with TrkA protein levels. ProNGF expression in CCI animals was significantly higher than in sham-operated controls from 1-4 weeks post-CCI. ProNGF immunoreactivity was increased in mast cells at 1 week post-CCI and, at later time points, in keratinocytes. P75 expression within the dermis and epidermis was significantly higher in CCI-operated animals than in controls and these changes were localized to neuronal and non-neuronal cell populations using specific markers for each.

**Conclusions:**

We describe proNGF expression by non-neuronal cells over time after nerve injury as well as the association of NGF-responsive fibers to proNGF-expressing target tissues. ProNGF expression increases following nerve injury in those cell types previously suggested to express it.

## Background

Nerve growth factor (NGF) is a 13 kDa neurotrophin [1]. Its roles within the peripheral nervous system include the maintenance of the adult sensory afferents and sympathetic post-ganglionic efferents [2,3]. During embryonic development, its expression is essential for the normal development and maturation of the sympathetic nervous system [4]. Mice engineered to over-express NGF in keratinocytes were associated with increased peptidergic fiber density as well as inappropriate innervation by sympathetic efferents [5,6]. These mice were also shown to have heightened sensitivity to applied heat and mechanical stimuli [7]. We have previously demonstrated that following nerve injury, there was an increase in sympathetic and peptidergic innervation in skin, comparable to that occurring following the overexpression of NGF [8-12]. We have also shown that an increase in sympathetic and peptidergic innervation occurs following application of nerve injury models [8-10]. These observed changes in NGF-responsive sympathetic and sensory fibers have been proposed to be mediated through the cell-surface NGF receptors, the high affinity receptor TrkA and the low-affinity pan-neurotrophin receptor p75 [13]. In agreement with this, TrkA and, to a lesser extent, p75 were detected in both post-ganglionic sympathetic efferents and peptidergic sensory afferents [14,15]. However, p75 was mainly expressed on Schwann cells, a cell type which is in direct contact with the nerve fibers [16]. Dimeric NGF binds its cognate receptor, TrkA and upon complex formation with p75, TrkA undergoes a conformational change enhancing its binding affinity for NGF [17-20]. The half-life of NGF binding to p75 is very rapid such that dissociation times have been estimated at < 3 sec [20-22]. NGF is synthesized by a variety of peripheral cell types including mast cells, lymphocytes, keratinocytes and vascular endothelium in its precursor form proNGF [23-28]. The receptor p75 has a high affinity for proNGF, unlike its mature form (mNGF), and when ligand bound, engages pro-apoptotic signaling cascades [29,30]. Keratinocytes have been demonstrated to express a number of ligand-gated ion channels such that upon ligand binding, cause membrane depolarization and release of a variety of factors including proNGF and ATP [31-33]. Within the extracellular space, proNGF is converted to its mature form, mNGF, through a protease cascade [34]. The enzymes involved in this cascade include plasminogen, which is converted to its active form, plasmin, by either tPa or uPa. Plasmin in turn converts proNGF into its mature form, mNGF, and also converts proMMP-9 to its mature form, MMP-9, which in turn degrades mNGF [34,35]. Previous literature describing the distribution of NGF in the periphery has been restricted to studies following injury, a point in which NGF is detectable within responsive nerve fibers [36] other than an early study examining the distribution of NGF in keratinocytes [27]. A clear differentiation between proNGF and mNGF could not be made in the above mentioned examples since the antibody used cannot differentiate the mature from the precursor form. Following nerve injury, Wallerian degeneration is triggered, a process necessary for those axons directly affected by the ligature and experiencing demyelination to be successfully cleared of myelin debris by recruited macrophages as well as for subsequent sprouting of uninjured axons [37]. During the process of Wallerian degeneration, macrophages and mast cells are recruited to the site of injury [38] where their phagocytic capabilities assist in clearing myelin debris [38]. Activation of local Schwann cells is followed by increased production of Il-1β and TNF-α, both of which increase transcription and translation of NGF within local cell types [23,39-41]. This increase in peripheral Il-1β and TNF-α takes on additional significance when it is considered that they can lead to increases in proMMP-9 protein levels as well as its conversion to the mature form within skin; it has also been shown that cell surface plasmin levels were also increased following the increase in Il-1β and TNF-α [42-44]. It has long been known which cell types are responsible for the production of NGF; however these studies have employed indirect methods such as culturing, *in situ *hybridization or over-expression systems [24,38,45,46].

NGF has been strongly implicated in both neuropathic and inflammatory pain conditions [47-51]. It has been shown that following activation of Schwann cells as part of the Wallerian degeneration response, Schwann cells dramatically upregulate p75 on their cell surface [16,52,53]. The exact role of this upregulation has not been clearly defined, although it has been proposed that p75 in this case acts as a sequestering molecule to keep the increased NGF on the cell surface, to facilitate its function as a chemo attractant molecule for regenerating peptidergic afferents [53,54].

The increase in available NGF associated with both neuropathic and inflammatory pain has been well documented [38,51,55-57]. Mice engineered to over-express NGF in keratinocytes not only develop hyperinnervation in the target tissue region by peptidergic afferents and sympathetic efferents but also display sympathetic basket formation around populations of dorsal root ganglia neurons [5,58].

Our lab has consistently demonstrated that following nerve injury there is a dramatic initial loss of myelinated and unmyelinated sensory afferents [8-10,12]. Four weeks following application of the chromic gut-based chronic constriction nerve injury, there was a return to sham operated control of peptidergic afferents followed by the development of a significant increase in peptidergic fiber density at later stages [8,12]. We also detected a sprouting of sympathetic efferents into the upper dermis, a territory from which they are normally devoid, and which not only innervated areas of the dermis away from blood vessels but associated very closely with peptidergic afferents [8].

We undertook the current study to test the hypothesis that, following nerve injury, the innervation changes of peptidergic afferents and sympathetic efferents follow a change in expression of proNGF in the periphery and that this is closely associated with Schwann cell production of proNGF.

## Results

### proNGF Distribution

**Figure 1:**
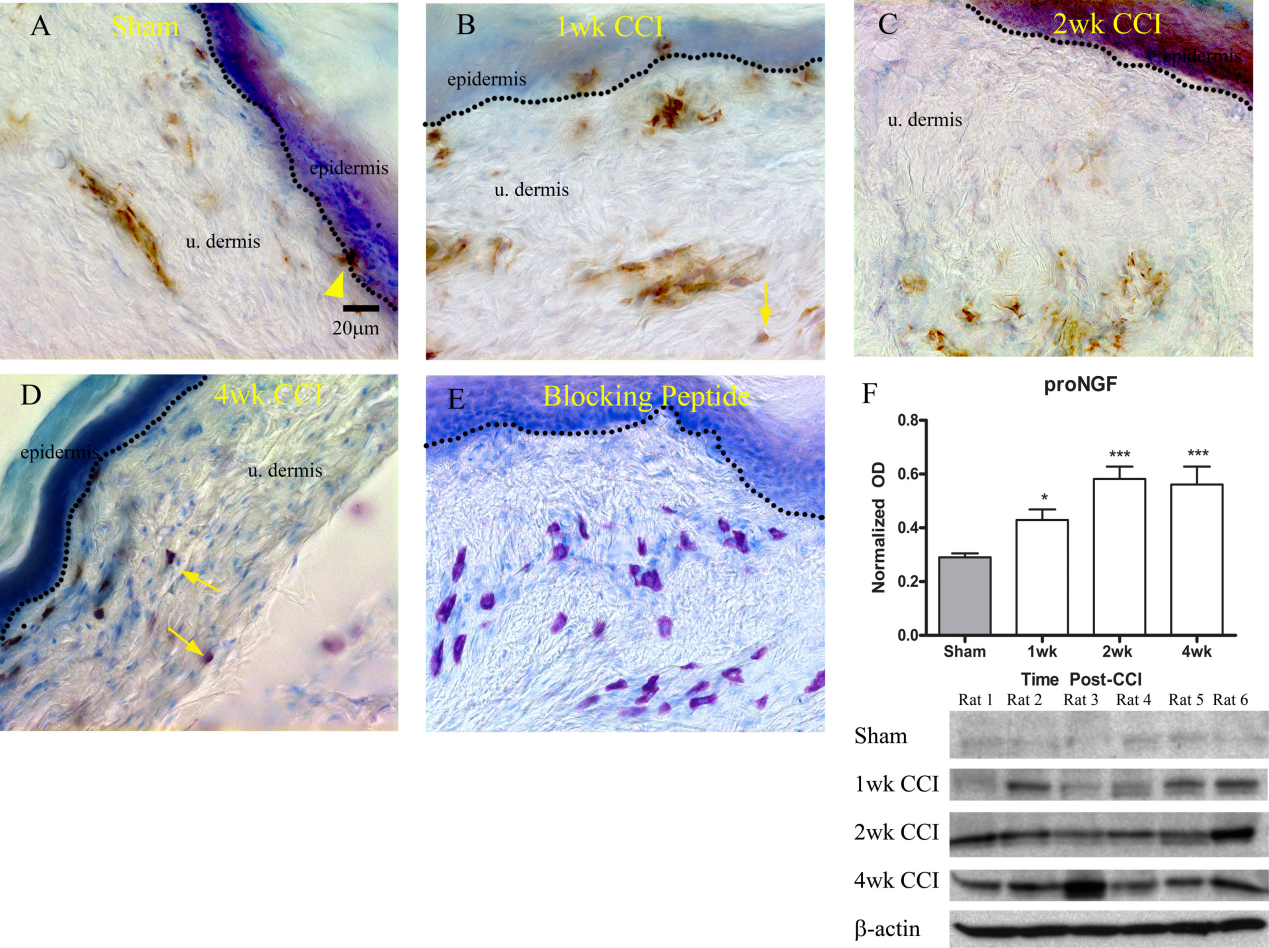
**Distribution of proNGF immunoreactivity in naïve glabrous skin and following application of CCI**. A) In sham operated controls, proNGF (brown precipitate) was found in the upper dermis, particularly in wall of blood vessels, and in small patches of keratinocytes (arrowhead). B) 1 week post-injury, proNGF immunolabeling was detected in mast cells (arrows; identified based on toluidine blue metachromatic counterstaining), in blood vessels in the upper dermis as well as in keratinocytes. C) At 2 weeks post-injury, note the intense immunostaining of keratinocytes, the labeling of some unidentified structures in dermis and a lower number of immunostained mast cells. D) At 4 weeks post-injury, note mast cell immunolabeling. E) Preincubation of antibody with control peptide abolished all specific staining. F) proNGF quantification was done by Western Blot analysis (n = 6) from sham, and animals 1 - 4 week post-injury. OD was normalized against β-actin loading controls. ***p < 0.001, **p < 0.01, *p < 0.05; +SEM.

To study the normal distribution of proNGF immunoreactivity, we used bright field microscopy (Figure 1A). proNGF immunoreactivity was detected in well defined but sparse patches as a brown DAB precipitate in the epidermis and dermis. In the epidermis, proNGF immunoreactivity was restricted to small patches of labeled keratinocytes along the dermo-epidermal junction (Figure 1A; arrowhead). Mast cells were identified based on metachromatic staining of heparin in granules using toluidine blue. In sections from control animals, mast cells were not abundant in the upper dermis and were only faintly immunoreactive for proNGF (not shown). proNGF was also found in the endothelium of capillaries and venules, identified based on the flattened appearance of the cells outlining the vessel lumen, and in perivascular cells (Figure 1A). One week post-CCI, the mast cell number qualitatively increased in the upper dermis and these cells were proNGF immunoreactive (Figure 1B; arrow). Two weeks following nerve lesion, mast cell infiltration in the upper dermis decreased, however proNGF immunoreactivity increased in other non-neuronal structures, including the vascular endothelium, fibroblasts and, particularly, keratinocytes (Figure 1C). The results at four weeks post-lesion were similar to those at two weeks, although labeling of keratinocytes was much less (Figure 1D**)**. Pre-incubation of the antibody with its control peptide abolished all specific staining (Figure 1E).

Western blots were done in order to more accurately quantify this change in expression levels of proNGF protein itself rather than indirectly through levels of intensity of bright-field images (Figure 1F). The sampled protein was taken from the same area of the glabrous skin in which immunohistochemistry was conducted. Care was taken during sample extraction and preparation to ensure no contaminating protein from cartilage or deep tissues such as striated muscle. Data from the Western blots demonstrate an increase in target tissue levels of proNGF at one week post-injury, with a further increase at two to four weeks (Figure 1F). These results taken together indicate that the increase in proNGF at one week post-injury is predominantly from infiltrating mast cells whereas at two weeks post-injury this increase in target tissue levels of proNGF has switched to keratinocytes and blood vessels.

**Figure 2:**
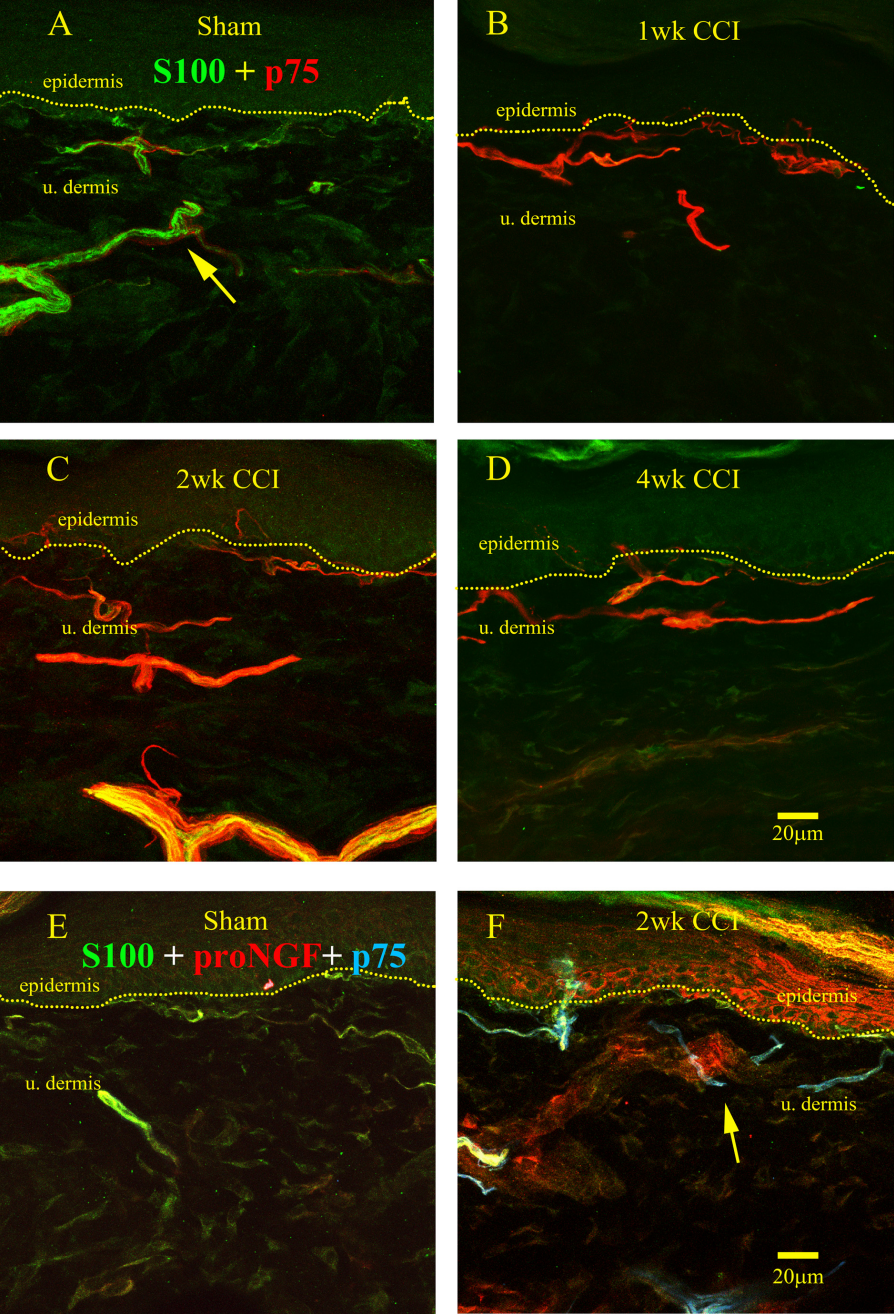
**Changes in p75 expression on S100-IR Schwann cells**. A) In sham-operated rats Schwann cells (green) were detected by means of S100 immunoreactivity in small nerves, with immunoreactivity of varying intensity from bright (arrow), lower in the dermis, to dim, along the dermo-epidermal junction. P75 immunoreactivity (red) was evident surrounding all S100-IR Schwann cells. B) One week following nerve injury, p75 immunostaining was dramatically upregulated in Schwann cells; the intense red color masked the mixture of red and green stainings which could be detected by analysing the separately the S100 and p75 stainings (not shown). C-D) 2 & 4 weeks post-injury a decrease in p75 intensity was observed in that the yellow indicative of S100 co-labelling was able to be visualized. E) proNGF (red) S100 (green) and p75 (blue) triple labelling to demonstrate the relative distribution of Schwann cells with respect to proNGF; note a limited distribution of Schwann cells with proNGF and faint immunoreactivity for p75 in sham-operated controls. F) 2 weeks post-injury, the clear upregulation of p75 immunoreactivity associated with S100 (arrow) was observed, which wrapped around proNGF-IR blood vessels.

**Figure 3:**
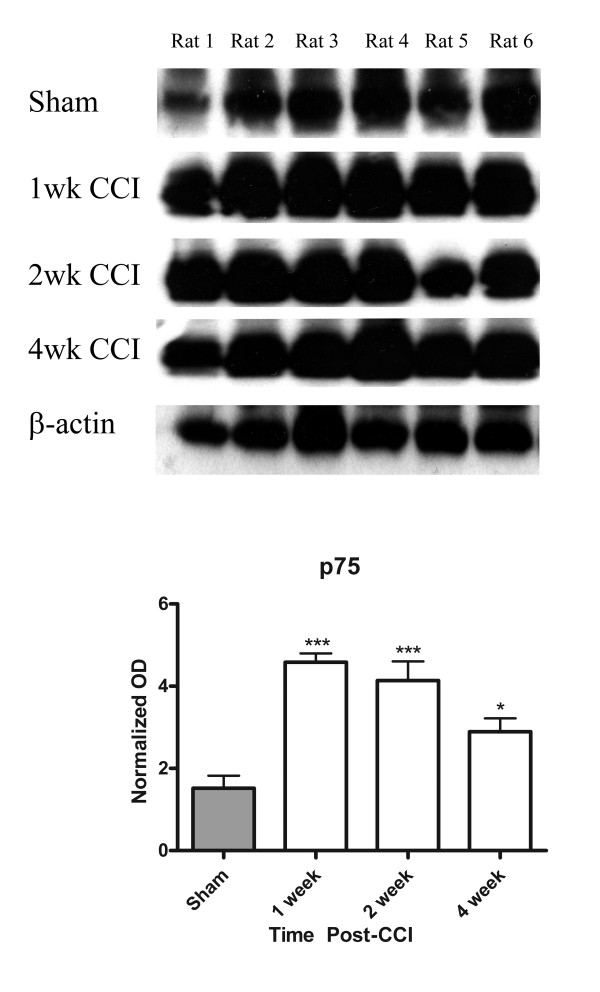
**Quantification of p75 protein content in glabrous skin**. Glabrous skin was taken from the same region sampled for immunocytochemistry. A single band corresponding to that of the PC12 cell culture supernatant used as positive control was observed. Note the increased p75 protein levels particularly at 1-2 weeks post-lesion. OD was normalized against β-actin loading controls. N = 6; ***p < 0.001, **p < 0.01, *p < 0.05; +SEM.

It has long been shown that p75 is expressed on the surface of Schwann cells and that this expression is upregulated following nerve injury [16]. To confirm and expand this observation, S100 was used as a marker for Schwann cells at the light microscopy level in a co-staining for p75 to show the distribution of the receptor on Schwann cells following nerve injury. In sham operated animals, S100 immunoreactivity was present with varying intensities depending on location (Figure 2A, in green). Thicker cutaneous nerves, further from the dermo-epidermal junction, were most intensely labeled, likely because they are rich in A-fibers surrounded by myelinating Schwann cells, in which S100 content is known to be high. We also observed some S100 immmunostaining, usually not very intense, along the dermo-epidermal junction which might represent non-myelinating Schwann cells associated with C-fibers, which are known to contain less S100 protein than myelinating Schwann cells [59]. P75 receptor immunoreactivity (in red) in sham-operated controls was low and often surrounded S100-IR structures, likely representing immunostaining in perineurial cells; it was also occasionally seen within the S100-IR structures, possible representing p75 expression on sensory axons (Figure 2A; arrow). One week following nerve injury (Figure 2B), the relative intensity of p75 to S100 changed dramatically as p75 immunoreactivity was significantly more intense than that of S100, correlating with the significant increase in protein content (see Figure 3). At two to four weeks post injury (Figures 2C-D), S100 and p75 immunoreactivities were both intense and heavily co-localized. The co-localization of the two immunoreactivities occurred at all time points studied post-injury. A triple labeling showing p75, S100 and proNGF immunoreactivities (Figure 2E-F) in sham-operated and at two weeks post-injury allowed us to show the distribution of each of these immunoreactive structures with respect to the other either before or after injury. It was found that in sham operated controls that p75 and S100 immunoreactivity are found within the same nerve fiber bundles, where S100 was more intense than that of p75. However, after injury, proNGF immunoreactivity increased in vascular endothelial-like structures and keratinocytes, whereas strong p75 immunoreactivity was seen in Schwann cells. Importantly however, a co-localization between p75 on Schwann cells and proNGF was not observed.

### Changes in NGF-Responsive Fiber Innervation

**Figure 4:**
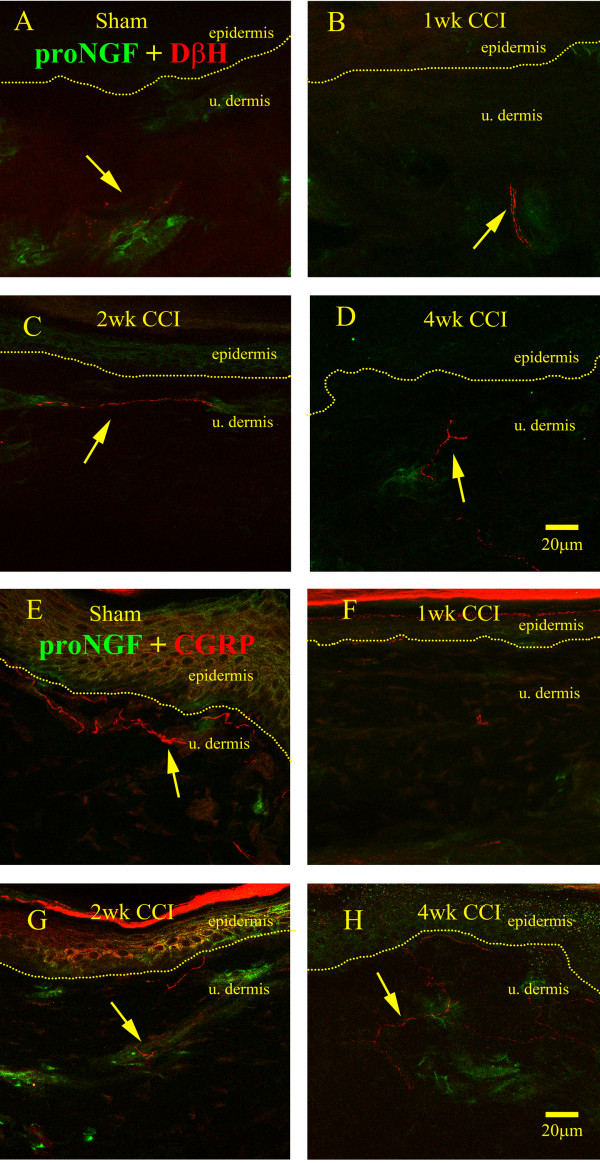
**Sympathetic and peptidergic nerve fibre association with proNGF**. A) DβH-IR sympathetic efferents (red) were closely associated with proNGF (green) in sham operated control animals. B-C) At 1 and 2 weeks post-injury, no detectable difference was observed in the pattern of sympathetic efferent distribution with respect to proNGF. D) At 4 weeks post-lesion sprouted sympathetic efferents retained their association with proNGF, and continued to sprout towards the dermo-epidermal junction. E) In sham-operated rats, peptidergic afferents were distributed along the dermo-epidermal junction in which the occasional group of proNGF immunoreactive cells was found. F) At 1 week following nerve injury, very few peptidergic afferents were observed. G) At 2 weeks post-injury, peptidergic afferents were found along the dermo-epidermal junction as well as along proNGF immunoreactive cells (arrow). H) At 4 weeks post-lesion, peptidergic afferents increased in numbers were loosely associated with proNGF-IR cells (arrow).

Due to the known cell surface expression of the high-affinity TrkA receptor and of p75 on both post-ganglionic sympathetic efferents and sensory peptidergic afferents, we sought to determine what relationship existed between proNGF producing cell types and these fibers in sham operated controls and after nerve injury (Figure 4). We observed that the post-ganglionic sympathetic efferent population, as detected by DβH immunoreactivity, seemed to innervate proNGF-IR structures (Figure 4A). In sham operated controls, the DβH-IR fibers appeared to wrap around the proNGF-IR cells in the wall of presumptive blood vessels. At one and two weeks post-injury, the association of DβH-IR fibers with proNGF producing cells remained similar to sham in that there was either a restriction of the DβH-IR fibers to the lower dermis (Figure 4B) or a close association between the DβH-IR fibers with blood vessels (Figure 4C). However, at four weeks post-injury, a time point when our lab has previously reported a significant presence of sympathetic efferents in the upper dermis away from blood vessels [8,9], we observed that DβH-IR fibers still innervated proNGF producing structures; however, the sections of the fibers closer to the epidermis were not associated with proNGF (Figure 4D). In sham-operated animals, the peptidergic, CGRP-IR afferents were also seen to have segments close to proNGF producing structures, but the association of the two was not as obvious as for the sympathetic (Figure 4E). At one week post-injury, a time point at which CGRP-IR afferent loss was maximal; there was no association between the remaining CGRP-IR fibers and proNGF-IR structures (Figure 4F). At two weeks post-injury, however, there was an obvious association between CGRP-IR fibers and proNGF producing cells (Figure 4G). Interestingly, at four weeks post-CCI, a time point at which peptidergic afferents returned to sham-operated levels [8,12], the association was not as close as that observed at two weeks (Figure 4H).

### P75 association with other structures

**Figure 5:**
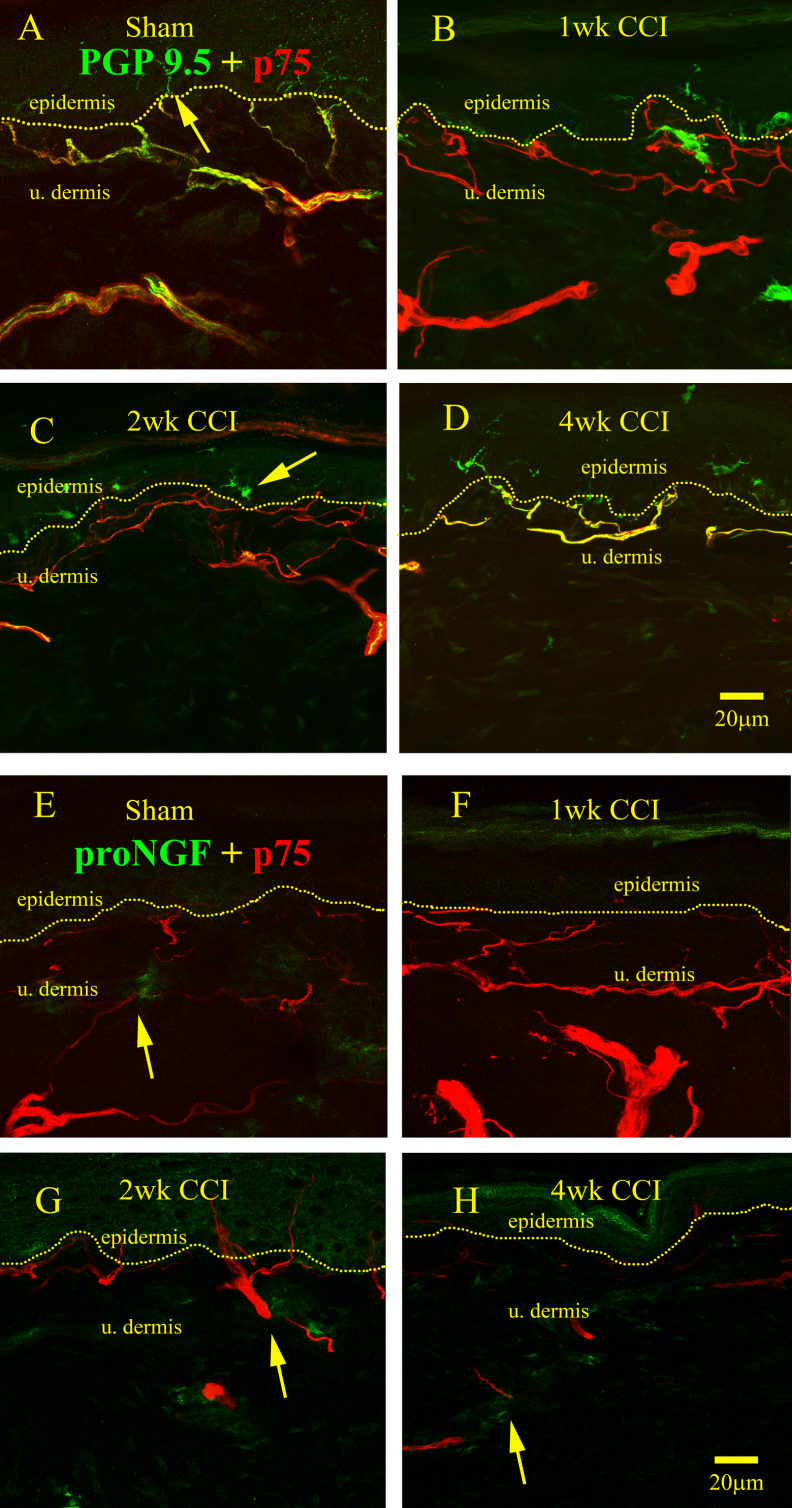
**Distribution of p75 immunoreactivity following nerve injury and relationship to nerve fibers and proNGF**. A) In sham-operated animals, p75 was distributed (red) around and with PGP 9.5-IR nerve fibers (green). P75 staining was found more clearly around large cutaneous PGP 9.5-IR nerve fiber bundles and smaller fibers along the dermo-epidermal junction. Where nerve fibers crossed the dermo-epidermal junction into the epidermis, the yellow color representing p75 associating with nerve fibers was lost (arrow). B) At 1 week post-injury, virtually all PGP-9.5-IR nerve fibers disappeared from the upper dermis and epidermis; immunostaining in p75-IR Schwann was very intense. C) At 2 weeks post-injury, a low number of PGP-9.5-IR fibers were detected and were associated with p75 Schwann cells (yellow), however most of the PGP-9.5-IR was restricted to Langerhans cells in epidermis (arrow) D) At 4 weeks post-injury, p75 immunoreactivity decreased co-incidentally with the increase in PGP-9.5 immunoreactivityin the upper dermis (yellow). E-H) ProNGF and p75 association in sham-operated controls and in lesioned animals was loose in that most proNGF immunoreactivity was segregated from that for p75 and there was no obvious co-localization (arrows).

We have also investigated the association of nerve fibers identified with the pan-neuronal marker, PGP-9.5, with p75 expression (Figure 5). In sham operated controls, PGP-9.5-IR nerve fibers were distributed throughout the epidermis and in the upper dermis and formed a dense plexus (Figure 5A). In the lower dermis, PGP9.5-IR fibers were found in small cutaneous nerves with a sheath of p75 immunoreactivity around them, likely representing staining of perineurial cells (Figure 5A). Interestingly, PGP9.5-IR fibers penetrating the epidermis were not associated with p75 immunoreactivity (Figure 5A). At one week post-nerve injury, there was scarce PGP-9.5 immunoreactivity in the upper dermis, consistent with previous reports of a dramatic decrease in nerve fiber innervation at this time point [12] (Figure 5B). However, p75 was strongly upregulated (Figure 5B), likely due to strong Schwann cell labeling (see Figure 2A). At later time points (Figure 5C-D), PGP9.5-IR fibers were progressively more abundant and were associated with p75 immunoreactivity. At one to four weeks post-injury, PGP-9.5-IR structures in the epidermis represented mostly Langerhans cells staining (Figure 5B-D), as nerve fibers were mostly depleted from the epidermis.

We also investigated the relationship of p75 with respect to proNGF immunoreactivity. We found that in the sham-operated controls (Figure 5E), proNGF-IR patches within the upper dermis loosely associated with p75-IR structures, however there was no colocalization as indicated by the absence of yellow color. At one week post-injury, the intensity of the p75 immunoreactivity greatly surpassed that of proNGF (Figure 5F), and at two and four weeks post injury, we were able to demonstrate sparse p75 immunoreactive fibers in the epidermis as well as in the upper dermis (Figure 5G-H).

### Western Blot

The results of quantification of by Western blot of the relative increase in p75 expression following nerve injury are illustrated on Figure 3. Consistent with the immunohistochemical data, there was a very significant increase in p75 expression at one week post-injury. The high protein levels persisted at two weeks post-injury, and were lower, although still elevated, at four weeks post-CCI (Figure 3). The changes in p75 protein levels were parallel to those seen using immunohistochemistry (Figure 2A-D & Figure 5).

**Figure 6:**
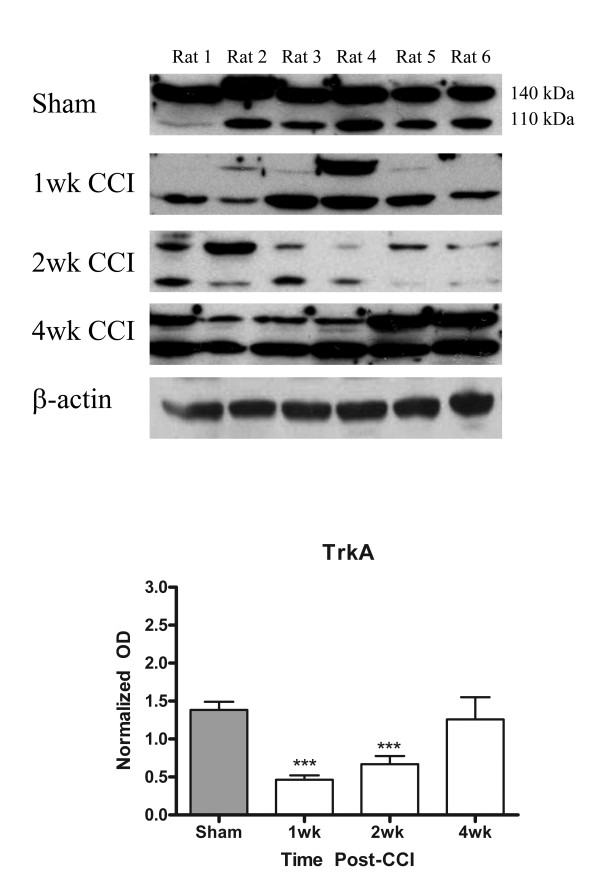
**Quantification of TrkA protein content in glabrous skin**. TrkA protein was sampled from glabrous skin of same region used for immunocytochemistry. Two bands were recognized by the monoclonal anti-TrkA antibody, a 110 kDa and 140 kDa form corresponding to the immature, unglycosylated form and mature form respectively. TrkA levels decreased significantly at 1 week post-injury and remained significantly lower at 2 weeks post-injury until 4 weeks, at which point no difference was observed compared to sham-operated controls. OD is normalized against β-actin loading controls. ***p < 0.001, **p < 0.01, *p < 0.05; +SEM.

The quantification of the changes in TrkA expression within the glabrous skin of the rat hind paw was done using Western Blot (Figure 6). We observed that a significant decrease in TrkA protein content occurred at one and two weeks following nerve injury with a recovery at four weeks post-lesion. This is consistent with the observation that peptidergic afferents retract from the upper dermis and epidermis shortly after injury and recover by four weeks [8,12].

## Discussion

In this study, we performed for the first time a detailed description of the relationship of the NGF-responsive nerve fiber populations to the neurotrophin-producing cells following the application of a commonly used neuropathic pain model, the CCI of the sciatic nerve. Because NGF is known to occur in cells in its precursor form [34], proNGF, we used antibodies specific for this precursor form.

Previous research has focused on the changes in mature NGF uptake following nerve injury given that mNGF is expressed at very low levels in target tissues in sham or naïve animals [36]. Methods such as *in situ *hybridization have shown that the mRNA for NGF is transcribed in Schwann cells [24,45] and fibroblasts [60] and that the protein exists in mast cells [26] and keratinocytes [27]. Cultured mast cells have also been demonstrated to upregulate NGF when presented with the cytokines IL-1β and TNF-α [39,40,61], and when degranulated release NGF into the culture media [26]. Schwann cell production of NGF has been demonstrated using culturing methods in which morphology of Schwann cells in explanted optic nerve has been shown to correlate with NGF expression [45]. A line of evidence that has pervaded research literature is that a chemoattractant gradient of NGF created by its retention through cell surface expressed low-affinity NGF receptors, p75 [53,62]. However, due to the restrictions of the available methodology, this has not yet been directly proven.

In the current study we provide some important new evidence, namely that a significant expression of proNGF by Schwann cells, identified by S100 immunoreactivity, was not evident. We also show an association of proNGF-producing cell types with NGF-responsive sensory and sympathetic fibers. Consistent with a number of prior observations, we were able to confirm the observed upregulation of p75 on non-neuronal cell types, namely Schwann cells, following nerve injury [16,23,63]. This upregulation was significant from early time points. Also, we have been able to correlate an immunocytochemical decrease in peptidergic innervation in the dermis and epidermis (the territories we sampled by Western blotting) with TrkA protein levels. Unfortunately, because of limitations of the availability of a reliable, commercially available anti-TrkA antibody for immunocytochemistry applications, we could not study the detailed anatomical distribution of TrkA immunoreactivity. TrkA levels in the skin can change due to a number of reasons, namely by a decrease in the level of expression in the cells or by a migration of the TrkA-expressing cells to other territories. Previous work from our lab and others demonstrate a retraction or 'dying back' of peptidergic and non-peptidergic nociceptive primary afferents from the epidermis and upper dermis [64]. This has been demonstrated by examining the distal regions of the common sciatic nerve following application of the CCI in which axons can be shown to degenerate, as well as by examining the upper dermis directly using retrograde tracers and pan-neuronal markers [12,65]. The results of these studies strongly suggest that the lack of peptidergic innervation is not due to a decrease in phenotypic markers such as receptor or peptide expression, but rather to fiber loss. It is also TrkA expression on the regenerating afferents which permits the regeneration to occur [66]. We have previously shown that peptidergic afferents return to innervation density of sham-operated controls by four weeks post-lesion, and that this recovery precedes that of any other fiber population [12].

This report provides further evidence that Schwann cells persist within the denervated territory. We have previously shown at the light and electron microscopic levels that following a complete transaction of a peripheral nerve, there was a complete loss of sensory fibers from peripheral nerves and that the denervated Schwann cells upregulate p75 in the absence of axon-Schwann cell contact [16]. Our present data expands on that observation by using a model in which there is a partial nerve lesion and some axons remain uninjured [67,68]. Indeed, in our material it was clear that from one week post-lesion Schwann cells upregulated p75 and at two weeks post-injury those Schwann cells surrounding either undamaged or regenerating nerve fibers labeled with the pan-neuronal marker PGP-9.5.

A surprising result from this study is the lack of a dramatic upregulation of proNGF levels in Schwann cells. Indeed, it has been suggested in many studies that, following nerve injury, Schwann cells would be among the key producers of NGF and it would be mostly the NGF from this source that would contribute to the sprouting of peptidergic afferents within the periphery [53]. One of our central premises is that cells immunoreactive for proNGF are sources of NGF for responsive nerves. Furthermore, that proNGF is released from the cells into the extracellular space in an activity dependent manner, and transformed into the active form, mNGF [34]. One possibility might be that we are detecting only proNGF and not mNGF, and that the latter form would occur on Schwann cells. We can exclude that possibility because mNGF occurs in very low concentrations even after injury, as detected by Western blot using an antibody that recognizes both proNGF and mNGF [69]. Another possibility would be that the turnover of proNGF in Schwann cells is very high, meaning that the relative lack of immunoreactivity could be indicative of rapid rate of release rather than a lack of production. Schwann cells, like keratinocytes, have been suggested to express ion channels such as P2X receptors [70] and sodium channels [71] and that upon activation depolarize the cell's membrane which could potentiate the release of neuroactive molecules such as proNGF into the intercellular space, but this does not explain why proNGF can be detected on keratinocytes and not in Schwann cells. However, based on the above, it is may also be possible that Schwann cells represent only a minor source of NGF.

We were also able to demonstrate a close association between proNGF producing cells and NGF responsive afferents from one to four weeks post-injury; these include sympathetic efferents and peptidergic afferents. There seemed to be a closer association between the sympathetic fibers and proNGF-IR cells than between peptidergic afferents and proNGF-IR cells. Historically it has been shown that sympathetic efferents, rather than sensory afferents are exquisitely responsive to changes in levels of NGF in the environment and this was demonstrated by neurite outgrowth when NGF was added to culture media [4,72]. Our observations that there was a closer association between the sympathetic efferents and proNGF producing cell types than for the peptidergic afferents may be reflective of the relative degree of dependence or sensitivity of each fiber population to NGF. It is thus not surprising that those structures which were more immunoreactive for proNGF were blood vessels (Figure 1B, 2F, 4C), which are known to receive dense innervation by sympathetic efferents [73]. The changes after lesion in proNGF production are consistent with the changes following the onset of Wallerian degeneration, as within one week following nerve injury we observed a large degree of mast-cell infiltration in the upper dermis (Figure 1B).

## Conclusions

In summary, we were able to show that following application of the traditional CCI model of neuropathic pain, mast cells followed by keratinocytes and vascular endothelium upregulate proNGF, and that this upregulation was significant until at least four weeks post-injury, as confirmed by Western blotting. This is in contrast to the relative changes in TrkA protein levels and p75 within the same area. Following nerve injury, there was a dying back of peptidergic afferents and a correlative decrease in TrkA protein until four weeks post-injury, at which point it returned to levels of sham-operated animals, coinciding with the recovery of the peptidergic innervation. Our data suggests that the sprouting after lesion of NGF-responsive fibers is initiated by the increase in local proNGF (and thus mNGF) levels, and may be maintained with the involvement of other factors.

## Methods

In total, 60 male Sprague-Dawley rats (Charles River Canada), weighing between 150-175 g, were used for this study. All animals were maintained on a 12 hour light/dark cycle and allowed access to food and water *ad libitum*. All animal protocols were approved by the McGill University Animal Care Committee and followed the guidelines of the Canadian Council on Animal Care and the International Association for the Study of Pain.

### Peripheral Nerve Lesions

Animals were randomly assigned to receive either a traditional chronic constriction injury (CCI) or sham operation. All animals were deeply anaesthetized with isoflurane. In total, 30 rats received the sham operation and 30 received the CCI injury to the common sciatic nerve, as described in detail by Bennett & Xie [74]. Briefly, the left common sciatic nerve was exposed at the mid-thigh through the biceps femoris. All surrounding fascia was removed to allow free passage to blunt glass probes. Proximal to the trifurcation of the sciatic nerve, four ligatures (4-0 chromic gut, Ethicon) were tied loosely around the nerve with ~1 mm spacing in between. The incision was closed in layers using absorbable sutures (Vicryl, Ethicon). The animals assigned to sham-operated groups underwent the above mentioned procedures with the exception of the application of the chromic gut ligatures.

### Animal perfusion and histological or Western blot processing

Animals were sacrificed after periods of one, two, and four weeks post-surgery. Rats were administered Equithesin (6.5 mg chloral hydrate and 3 mg sodium pentobarbital) in a volume of 0.3 mL, i.p., per 100 g body weight. Three animals per time point were perfused for immunohistochemical analysis. Once animals were deeply anesthetized, they were quickly transcardially perfused with vascular rinse (0.1% w/v sodium nitrite in a phosphate buffer) followed by histological fixatives (3% paraformaldehyde, 15% saturated picric acid in 0.1 M phosphate buffer pH 7.4) for 30 minutes. The glabrous skin from the left hind paw, specifically the thin skin surrounded by tori, was removed, post-fixed for one hour in the same fixative used for the perfusion and placed in 30% sucrose in 0.1 M phosphate buffer for cryoprotection overnight at 4°C. Tissue was then embedded in an optimum cutting temperature medium (OCT, TissueTek), cut using a cryostat (Leica) at a thickness of 50 μm and processed as free floating sections. A second cohort of six animals per time point were perfused for 30 seconds with ice cold saline to clear contaminating blood. The same region of glabrous skin as that used for immunohistochemistry was excised from the rat hind paw, snap frozen in liquid nitrogen and stored at -20°C for no more than ten days until processed for Western blot analysis.

### Immunohistochemistry

Sections were washed in PBS for 30 minutes. In all subsequent steps, phosphate buffered saline (PBS) with 0.2% Triton X-100 (PBS-T) was used to dilute the immunoreagents and for washing. Sections were treated with 50% ethanol for 30 minutes followed by one hour incubation in PBS-T containing 10% (v/v) normal serum of the species used to generate the secondary antibody to reduce non-specific staining. Subsequently, sections were incubated with primary antibodies (diluted in PBS-T) at 4°C overnight (or 48 hours in the case of S100). The following primary antibodies were used: polyclonal guinea-pig anti-human calcitonin gene related peptide (CGRP; 1:4000, Bachem; Torrance CA), monoclonal mouse anti-dopamine β-hydroxylase (DBH; 1:5 MediMabs, gift of Dr. A. Claudio Cuello), polyclonal rabbit anti-proNGF (1:500, Alomone Labs, Israel), monoclonal mouse anti-p75 (MC192; 1:5, Novus Biologicals), polyclonal rabbit anti-S100β (1:5000; Swant Switzerland) and polyclonal rabbit anti-PGP 9.5 antibody (1:800, Cedarlane; Burlington Ont.). To assess by immunohistochemistry the specificity of the anti-proNGF antibody, we pre-adsorbed it with the control antigen supplied by Alomone (3 μg of control peptide to 0.8 μg of antibody) for three hours over ice prior to incubation with control tissue from naïve rats. After washing, the sections were incubated with secondary antibody diluted in PBS-T for two hours at room temperature. The following secondary antibodies were used: a) for those antibodies raised in mouse, a highly cross-adsorbed goat anti-mouse IgG conjugated to Alexa 596 (1:800, Molecular Probes, Eugene, OR); b) for those raised in rabbit or guinea-pig, a goat anti-rabbit or anti-guinea-pig conjugated to Alexa 488 (1:800, Molecular Probes, Eugene, OR); c) in the case of the triple labeling a highly cross-adsorbed donkey anti-mouse conjugated to Cy-5 (1:800, Molecular Probes, Eugene, OR). Sections were washed and mounted on gelatin-coated slides and coverslipped using Aquapolymount (Polysciences; Warrington, PA). In the case of S100 immunostaining, an hour incubation with an goat anti-rabbit biotin conjugated secondary (1:400; BA-1000; Vector) was used. Following this, sections were incubated for one hour with the A and B reagents (1:400 Vectastain ABC kit; Vector) followed by a two hour incubation with a streptavidin antibody conjugated to Alexa 488 (1:200; Molecular Probes, Eugene, OR). For the S100/p75/proNGF triple labeling, the anti-proNGF antibody was added following the completion of the staining with S100 and p75. In the case of bright field images using anti-proNGF antibodies, sections were prepared as described above except for a ten minute incubation with 0.1% H_2_O_2 _prior to blocking and following incubation with primary antibody, incubated for two hours with a biotinylated goat anti-rabbit secondary antibody (1:200, Vector). The sections were washed with PBS and incubated for one hour with the A and B reagents (4 μl of A + 4 μl of B per mL of PBS, ABC Vectastain Elite kit). The sections were then washed in PBS and incubated for 5 minutes in a solution of 3-3'-diaminobenzidine (Sigma, 5 mg for 10 ml of PBS-T). Hydrogen peroxide was then added to the solution to reach a final concentration of 0.01%. The reaction was terminated after ten minutes by the addition of PBS. The sections were washed in PBS, mounted on gelatin-subbed slides and air dried. Slides were rehydrated and briefly exposed to a 1% solution of toluidine blue, rinsed and dehydrated in ascending alcohols and xylene, then coverslipped with Entellan (EMD, Gibbstown, NJ).

### Primary Antibody Characterization

CGRP: The anti-CGRP antibody raised in guinea pig was purchased from Bachem (T-5027, lot A00098-2). It was generated against a synthetic peptide from human α-CGRP with the following sequence: H-Ala-Cys-Asp-Thr-Ala-Thr-Cys-Val-Thr-His-Arg-Leu-Ala-Gly-Leu-Leu-Ser-Arg-Ser-Gly-Gly-Val-Val-Lys-Asn-Asn-Phe-Val-Pro-Thr-Asn-Val-Gly-Ser-Lys-Al a-Phe-NH2. The antibody has 100% reactivity with human and rat α-CGRP, human CGRP (8-37), chicken CGRP, and human β-CGRP. It has 0.04% crossreactivity with human amylin and 0% crossreactivity with rat amylin and with human and salmon calcitonin as determined by radioimmunoassay (manufacturer's technical information). Although Western blot information was not available, the immunostaining has been localized in the skin to IB4 negative C-fibers, and fibers innervating blood vessels in rats [75,76] and in monkeys [77].

DβH: The anti-dopamine β-hydroxylase monoclonal antibody was received as a gift from Dr. Claudio Cuello (clone DBH 4; MediMabs, Montreal, Canada). It was generated against rat purified DBH. The specificity was tested by Western blot, ELISA and immunocytochemistry and it was found that it is highly specific for rat DβH, and does not cross-react with DβH in mouse, human, rabbit, bovine, guinea pig or cat tissues [78].

S100β: A rabbit polyclonal antibody purchased from Swant (Code Number 37A). It was generated against purified bovine brain S100β and shows < 0.5% cross-reactivity with S100α and reacts with S100β from all species (i.e. bovine, rat, chicken and human) (data obtained from supplier). This antibody has been used as a marker for Schwann cells [79].

P75: The monoclonal antibody against the low-affinity neurotrophin receptor (MC192, Novus Biologicals, Littleton, CO) was generated from solubilized rat PC12 cell membranes and was found to be selective towards rat p75 to the exclusion of gerbil, hamster, guinea pig or mouse p75 [80]. Its distribution in rat skin was described at both light microscopic and electron microscopic levels [16]

TrkA: Monoclonal anti-TrkA (R&D Systems MAB1056 Clone: 315104 Lot #WPO0108021) raised against recombinant rat TrkA extracellular domain (a.a. 33-418). This antibody recognized rrTrkA by ELISA and Western Blot with no cross-reactivity towards recombinant human TrkA, TrkB, TrkC, recombinant mouse TrkB and TrkC (information provided by supplier).

PGP 9.5: The rabbit anti-PGP 9.5 antibody (Ultraclone code RA95101) was generated by repeated injection of purified whole human PGP 9.5 in Freund's adjuvant into rabbits. The antibody cross-reacts with PGP 9.5 protein in all mammalian species (manufacturer's technical information). By Western blot, it recognizes a band at 38 kDa from human and rat skin, and preadsorption with purified PGP 9.5 completely abolished this recognition [81,82]. Immunohistochemical staining with this antibody in either rat or human skin was absent following preadsorption with purified human PGP 9.5 protein [81,82].

proNGF: The rabbit anti-proNGF antibody (Alomone #ANT-005 Lot#AN-03) was generated by injection of a synthetic peptide corresponding to a.a. 84-104 of the precursor form of rat NGF. It cross-reacts with rat, mouse and human proNGF (information from supplier).

### Western Blot

Samples were kept at -20°C until manually homogenized with liquid nitrogen and added to RIPA buffer (1% NP-40, 1% sodium deoxycholate, 0.1% sodium dodecyl sulfate, 150 mM NaCl, 25 mL Tris-HCl, pH 7.6) containing protease inhibitors (Complete, Roche Molecular Biochemicals, Indianapolis, IN). Samples were agitated overnight at 4°C, followed by 45 minute centrifugation at 13,200 rpm to separate supernatant. The supernatant was pipetted and protein quantified using Bio-Rad DC Protein Assay (Bio-Rad, Mississauga Ont). Samples were then prepared as either reduced (in the case of TrkA and proNGF) or not (in the case of p75) using 10% β-mercaptoethanol (Bio-Rad, Mississauga Ont) SDS-based protein loading buffer. Samples were then boiled for 5 min and 50 mg total protein for p75 westerns and 75 mg total protein for proNGF and TrkA westerns were loaded onto a 4% stacking and 12% separating acrylamide gel for SDS-PAGE at 90 V. Samples were transferred to nitrocellulose membranes for 90 minutes at 110 mA and blocked for one hour. Primary antibodies such as mouse anti-TrkA (1:500, MAB1056, R&D Systems, Minneapolis, MN), rabbit anti-proNGF (1:500, ANT-005, Alomone Labs, Isreal), or mouse anti-p75 (1:200, MC192, Novus Biologicals) were incubated overnight in blocking buffer at 4°C. The following day, membranes were washed with tris-buffered saline with 0.1% Tween-20 (TBS-T) and incubated for two hours at room temperature with secondary antibodies raised against either mouse (Product no.: 715-035-151) or rabbit (Product no.: 711-035-152) IgG, conjugated to horseradish peroxidase (1:5000, Jackson ImmunoResearch, West Grove PA), and incubated for one minute with ECL substrate (Western Lighting, Perkin Elmer, Montreal, Qc). Following x-ray film exposure, membranes were incubated for one hour with stripping buffer, blocked and incubated with mouse anti β-actin primary antibody (1:400, A5441, Sigma) in blocking buffer for one hour washed with TBS-T and incubated with anti-mouse IgG-HRP (1:5000). Immunoreactive bands were quantified by simultaneously obtaining equally sized bands and quantification was based on optical density and normalized against the loading control (β-actin) using MCID Image Analysis software (MCID4 Image Analysis System; Imaging Research Inc.; St Catherine's ON, Canada).

### Images for the Figures

Pictures for the figures of this publication were taken as Z-stacks of confocal optical sections using a Zeiss LSM 510 confocal microscope equipped with argon and helium neon lasers applying a 40X water-immersion objective. Images were originally saved in the Zeiss format, then exported directly into the TIFF format and adjusted for brightness and contrast only, using Adobe Photoshop CS4. Bright field images were taken using a Zeiss Axioplan 2 imaging microscope equipped with a 40 × Plan-Fluotar oil-immersion objective. Images were acquired with a high-resolution color digital camera using the Zeiss AxioVision software. Representative images of Western Blots were taken using an Epson scanner, saved as TIFF format and realigned using Adobe Photoshop CS4.

## Competing interests

The authors declare that they have no competing interests.

## Authors' contributions

JP designed and performed all experimental protocols described in this manuscript as well as the writing of the initial draft of the manuscript. ARdS provided supervision for data analysis, study direction, image acquisition, manuscript design and revisions. Both authors have read and approved the final draft of this manuscript.
